# Major coagulation disorders and parameters in COVID-19 patients

**DOI:** 10.1186/s40001-022-00655-6

**Published:** 2022-02-15

**Authors:** Azadeh Teimury, Mahshid Taheri Khameneh, Elahe Mahmoodi Khaledi

**Affiliations:** 1 Department of Cell and Molecular Biology, University of Kashan, Kashan, Iran; 2 Center of Excellence for Biodiversity, Faculty of Natural Sciences, University of Tabriz, Tabriz, Iran

**Keywords:** Anticoagulant therapies, Coagulation parameters, Coagulopathy, COVID-19, Thromboembolic events

## Abstract

Coronavirus disease 2019 (COVID-19), with a high prevalence rate, has rapidly infected millions of people around the world. Since viral infections can disrupt the coagulation and homeostasis cascades, various inflammatory and coagulation problems occur due to COVID-19 infection, similar to coronavirus epidemics in 2003 and 2004. According to multiple previous studies, in the present research, we reviewed the most commonly reported problems of COVID-19 patients, such as venous thromboembolism, pulmonary embolism, disseminated intravascular coagulation, etc. and investigated the causes in these patients. Coagulation and inflammatory markers, such as platelets and fibrinogen, C-reactive protein, lactate dehydrogenase, d-dimer, prothrombin time, etc., were also discussed, and the treatment options were briefly reviewed. In addition to coagulation treatments, regular examination of coagulation parameters and thrombotic complications can be helpful in the timely treatment of patients. Therefore, it is helpful to review the coagulation problems in COVID-19 patients. Although all mentioned problems and markers are important in COVID-19, some of them are more valuable in terms of diagnosis and prognosis.

## Introduction

COVID-19, which was first identified in December 2019 in Hubei Province, China, has caused a global pandemic [1]. Because of its similarity to severe acute respiratory syndrome (SARS), it is also referred to as SARS-CoV-2 in many studies [2]. Coagulation disorders are common in COVID-19 and are associated with the severity of the disease. With the incidence of a viral disease following inflammatory responses, an imbalance in pro-coagulant and anticoagulant mechanisms occurs, with endothelial dysfunction playing a major role [3]. COVID-19 patients with a history of coagulation problems worries about more risks than other patients [4].

Vascular damage during illness can increase the risk of thrombosis and cause disorders such as microvascular thrombosis and hemorrhage linked to extensive alveolar inflammation in patients with COVID-19 [5]. Researchers around the world are now seeking to minimize the problems caused by this disease. Overall, blood coagulation abnormalities in patients with COVID-19 can be considered as a prognostic factor [6]. Due to the severity of this disease, the levels of coagulation indicators, such as fibrinogen degradation products, lymphocytes, d-dimer, and accumulated platelets, may change in patients; therefore, examination of these factors can be used for a timely treatment. This review summarizes major coagulation disorders and parameters in COVID-19 patients and try to update scientific information about coagulation abnormalities and improve existing approaches for diagnosis and management of these disorders in COVID-19 patients.

## Coagulation problems

### Pulmonary embolism (PE)

Pulmonary embolism has been observed in many patients with COVID-19, which can lead to complications such as pulmonary vascular thrombosis and is associated with mortality risk [7].

In one study, autopsy of lungs from patients who died from COVID-19 and those from acute respiratory distress syndrome secondary to influenza infection were examined. The prevalence of extensive thrombosis and alveolar capillary microthrombi was significantly different in patients with COVID-19 [8]. ARDS, characterized by overactive coagulation system and inflammation, can increase the vulnerability of pulmonary embolism manifestations [9]. However, in one study, there was no significant difference in pulmonary embolism risk between patients [10].

D-dimer level determination is also used for the detection of pulmonary embolism. For example, in a study, d-dimer levels were high in people with pulmonary embolism and showed that the level of d-dimer can be considered as a predictor of pulmonary embolism [11] (see all factors in Table 1).

**Table 1:** Summary of coagulation problems plus the levels of coagulation parameters and inflammatory markers in patients with COVID-19

Group	Name	Abbreviation	Dominant changes	References
Coagulation problems	Arterial thromboembolism	ATE	Blood clotting	[26–31]
	Deep vein thrombosis	DVT	Blood clotting	[20–25]
	Disseminated intravascular coagulation/sepsis-induced coagulopathy	DIC/SIC	Blood clotting	[26, 32–40]
	Pulmonary embolism	PE	Blood clotting	[7–11]
	Venous thromboembolism	VTE	Blood clotting	[12–19]
Coagulation parameters	D-dimer	–	Increase	[57, 67–70]
	Factor VIII/Von Willebrand factor	FVIII/VWF	Increase	[71–77]
	Fibrinogen and Fibrin degradation products	FDP	Increase	[57–66]
	Lymphocyte count	–	Decrease	[41–46]
	Partial thromboplastin time/Prothrombin time	PTT/PT	Get longer	[64, 78–85]
	Platelet count	–	Decrease	[47–52]
Inflammatory markers	Antiphospholipid antibodies	aPL antibodies	Increase	[102–105]
	C-reactive protein/procalcitonin	CRP/PCT	Increase	[31, 88–95]
	Lactate dehydrogenase	LDH	Increase	[96–101]
	Neutrophil-to-lymphocyte ratio	NLR	Increase	[65, 66, 86, 87]

### Venous thromboembolism (VTE)

Venous thromboembolism is often clinically silent and in many cases, the first signs is a sudden fatal pulmonary embolism [12]. Complications of it occur frequently in hospitalized COVID-19 patients, which can also lead to pulmonary embolism and deep vein thrombosis [13]. At Union Hospital in China, the prevalence of venous thromboembolism was studied in 81 patients with severe novel coronavirus pneumonia (NCP). The increase in d-dimer levels was associated with the prevalence of venous thromboembolism and the value of this index decreased using anticoagulant therapies [14]. Mechanically ventilated patients have shown a high prevalence of VTE [15]. Due to the high prevalence of VTE in patients with COVID-19, systematic screening has been suggested in duration of illness [16]. In addition, relationship between Incidence of venous thromboembolism and bleeding in COVID-19 patients has been reported [17].

The prevalence of venous thromboembolism has been shown to be lower in COVID-19 patients who used prophylactic anticoagulants with anticoagulant therapy [18]. Long-term prevention with rivaroxaban has been shown to reduce the incidence of venous thromboembolism in patients [19].

### Deep vein thrombosis (DVT)

Similar to venous thromboembolism, deep vein thrombosis has also reported in COVID-19 patients in which blood clotting occurs deep in the veins [20]. The high prevalence of deep vein thrombosis and its association with adverse outcomes of COVID-19 has been reported in patients [21]. If coagulation problems occur early in COVID-19 disease, the prevalence of deep vein thrombosis may be asymptomatic and be associated with poor prognosis [22].

The relationship between deep vein thrombosis and d-dimer levels in COVID-19 patients was assessed. The results showed a higher level of d-dimer in patients with DVT and it can be used as a diagnostic capacity for it [23].

Data from 1783 patients with deep vein thrombosis in the critical stage showed that the diagnosis of it could play a role in the duration of hospitalization and ICU [24]. Due to the high prevalence of deep vein thrombosis in COVID-19, the assessment of this factor may be effective in the timely initiation of anticoagulants [25].

### Arterial thromboembolism

Arterial thromboembolism is a blockage in an artery that may extend to distant organs and occurs in patients with COVID-19 [26]. In a study, high number of venous thromboembolism events and arterial thromboembolism were recorded in patients from the beginning of admission [27]. Arterial thromboembolism is an obstruction that occurs under SARS-CoV-2 hyperinflammatory conditions and is a risk factor for patients [28].

A study showed that thromboembolic events associated with acute arterial ischemia and may lead to limb loss and even death [29]. It has been shown that arterial thromboembolism and venous thromboembolism was occurred despite a high utilization of thromboprophylaxis [30]. Although another study reported that the risk of mortality is reduced, if arterial thromboembolism and venous thromboembolism in patients of 60 years or younger treated properly [31].

### Disseminated intravascular coagulation (DIC)/sepsis-induced coagulopathy (SIC)

In some COVID-19 patients, the disease may become worse and septic shock may occur. Endothelial damage and failure of several organs due to coagulation problems are the results of sepsis, followed by disseminated intravascular coagulation [32]. It can be one of the changes caused by coagulopathy that appears in COVID-19 patients [33]. If blood coagulation increases, the platelets count will decrease, leading to disseminated intravascular coagulation. Then, exhaustion factors may arise due to the disruption in the synthesis of coagulation proteins and eventually, may result in bleeding [34, 35]. However, disseminated intravascular coagulation in some cases was rare [26].

The evaluation of the sepsis-induced coagulopathy is a great value in predicting the severe consequences of COVID-19 [36]. Sepsis-induced coagulopathy is a scoring system for coagulation disorders. It was found that diagnosing with sepsis-induced coagulopathy and disseminated intravascular coagulation could be helpful in prevention and treatment [37]. Of course, it cannot be declared with certainty that which one (DIC or SIC) occurs first [38].

The therapeutic effects of anticoagulants have been investigated in patients with sepsis. The criteria for this study were based on the disseminated intravascular coagulation and these drugs reduced mortality in patients [39].

Finally, nafamostat mesylate can be effective in reducing coagulation problems and has been reported to have the potential to reduce these problems [40].

## Coagulation parameters

### Lymphocyte count

It has been reported that in severe COVID-19 patients, there is a significant relationship between an increase in leukocytes and a decrease in lymphocytes. In addition, It has been reported that lymphocyte count was greater in younger people [41]. Changes in lymphocyte count is associated with oxygen demand [42].

Lymphocytopenia is common among patients with COVID-19 and significant decrease in the number of lymphocytes in a meta-analysis was shown in severe COVID-19 patients and it was found that the risk of exacerbation was three times higher in the presence of lymphocytopenia in patients [43]. In addition, coagulation disorders and hematological changes have been shown to be associated with decreased lymphocyte content in patients with COVID-19 [44]. In a retrospective study, the clinical data of COVID-19 patients were evaluated for lymphocytopenia. The results showed a low lymphocyte count and the occurrence of lymphocytopenia in ICU patients. Lymphocytopenia can also lead to acute kidney damage [45].

**Fig. 1:**
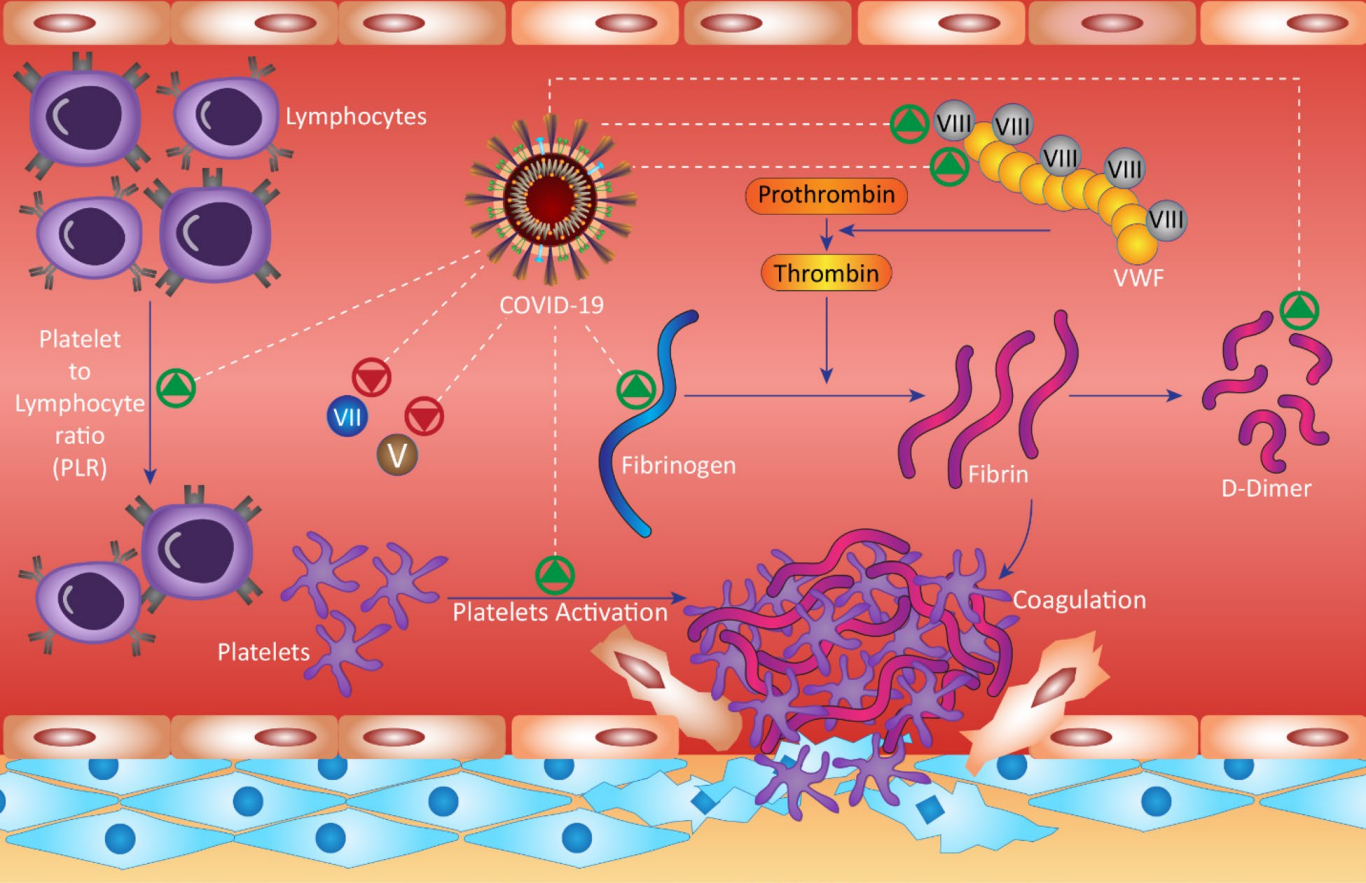
Coagulation markers involved in COVID-19. Green arrows mean an increase in the desired marker in patients, and red arrows indicate a decrease

Thymosin alpha 1 (Tα1) is a thymic peptide which has been effective to increase the number of T cells during the onset of severe lymphocytopenia in patients with COVID-19 [46]. These markers are shown in Fig. 1.

### Platelets count

Platelets is one of the important parameters in coagulation. Examination of the altered platelet gene expression in 41 patients showed that SARS-CoV-2 infection was associated with platelet hyperactivity [47]. In severe COVID-19 patients, an increase in platelet activation has been reported, whereas it does not occur in patients with mild disease and may participate in thrombus formation [48]. In addition, Lack of regulation of precoagulation platelets can play a role in the development of thrombotic complications caused by SARS-CoV-2 [49].

It was found that platelet to lymphocyte ratio (PLR) was an influential factor in severe COVID-19 patients and those with higher platelet to lymphocyte ratio had longer duration of illness [50]. In a study, there was a threefold increase in mortality in patients with COVID-19 with thrombocytopenia compared to those without it. Changes in platelet count were also associated with mortality [51].

The platelet adhesion molecule CD226 is expressed on platelet membranes and deletion of CD226 gene in the murine model has been shown to lead to increased platelet counts and enhanced platelet aggregation. Therefore, CD226 is considered as a candidate for the treatment of thrombotic disorders [52].

#### Thrombocytopenia (TCP)

Platelet count data of COVID-19 patients indicate that hospitalized patients have thrombocytopenia [51]. Thrombocytopenia characterizes by the destruction or reduction of platelets. It has been suggested that SARS-CoV-2, by inhibiting hematopoiesis through CD13 receptors, can decrease the initial platelet formation, reduce large numbers of blood cells, and lead to thrombocytopenia [53]. Data from 1476 patients with COVID-19 showed that thrombocytopenia increased the risk of mortality [54].

In addition, immune thrombocytopenia occurs in COVID-19 patients that is a bleeding disorder in which platelets become coated. In a study, patients with immune thrombocytopenia received thrombopoietin receptor agonists (TPO-RAs). The results in most cases showed, due to premature thrombocytosis, treatment should be adjusted or discontinued, which reduced immune activity can justify this action [55]. When using anticoagulants, one point must be considered. Heparin is an anticoagulant drug and it could decrease thrombotic complications in COVID-19 patients [26]. Adverse effects may occur in patients. One of these complications is heparin-induced thrombocytopenia that can be associated with venous and arterial thrombotic events [56].

### Fibrinogen levels and Fibrin degradation products (FDPs)

It has shown that fibrinogen level increases early in COVID-19 and can be used as a marker for early diagnosis of the disease [57]. In balance changes in fibrinolytic and coagulation pathways, damage to the endothelial membrane and pulmonary arteries causes the accumulation of coagulation factors, such as fibrinogen in the alveoli. Under these conditions, tissue factor (TF) causes fibrin formation [58].

A study was showed a high fibrinogen-to-albumin ratio that considered as an independent risk factor in disease progression [59]. Fibrinogen is more likely to reflect the acute phase than the thrombotic risk [60].

It has shown that, fibrinogen, and fibrin degradation products are high in patients with COVID-19-induced ARDS [61]. It has been reported that fibrin degradation products can predict mortality in COVID-19 patients and it may be argued that with the increase in d-dimer, the intrinsic fibrinolysis is developed in the lungs, resulting in bleeding due to the severe inflammation [62, 63].

A study showed that elevated fibrin degradation products was common in COVID-19 patients who died from NCP [64]. In addition, there is difference in this parameter between critical COVID-19 patients and non-critical [65]. Another study of 466 patients showed high level of fibrin degradation products with increasing severity of the disease [66].

### D-dimer

D-dimer is a fibrin degradation product that can help with blood clots. There is a relationship between changes in the d-dimer level and the prognosis of COVID-19 [23]. One study showed that d-dimer levels were high in critical patients that increasing the risk of thrombotic factors. In fact, the rate of thrombosis and bleeding in patients can be associated with a critical stage [67]. Data from one study showed high levels of d-dimer in hospitalized patients in ICU and patients who died. Progressive lymphopenia was also observed in them [68].

There is a relationship between increased d-dimer levels and mortality [69]. Clinical outcomes from two groups of COVID-19 patients and bacterial pneumonia were compared. The results showed high d-dimer levels in both groups at admission but higher levels in COVID-19 patients. They also found that d-dimer levels were associated with inflammatory markers, such as high sensitivity C-reactive protein (hsCRP) [70]. It is necessary to mention that d-dimer levels decreased before bleeding in COVID-19 patients [57].

### Factor VIII (FVIII) and Von Willebrand factor (VWF)

Factor VIII has been reported to be associated with the onset of thrombosis in patients [71]. Changes in the level of these factors disturb the balance of procoagulant and anticoagulant factors. Endothelial damage, can lead to the release of prothrombotic mediators, such as Von Willebrand factor. A disintegrin and metalloprotease with thrombospondin 1 repeats, number 13 (ADAMTS13) is a zinc-containing metalloprotease that cleaves Von Willebrand factor [72]. A study showed imbalance between alteration of the VWF‐ADAMTS13 axis and elevated Von Willebrand factor antigen to ADAMTS13 activity ratio COVID‐19 patients that enhances risk of micro thrombosis and hypercoagulation [73]. Thrombin, which is a trypsin-like serine protease, activate V and VIII factors that help the propagation of the coagulation process [74].

In one study, factor VIII activity was reported to be higher than normal in severe COVID-19 patients, while factor V and VII activity were lower [75]. It was also found that Von Willebrand factor antigen concentrations were higher than normal in COVID-19 ICU patients. Mortality is also associated with the Von Willebrand factor antigen [76]. It was suggested that monitoring the level of these factors could be effective in classifying the thrombotic risk [77].

### Prothrombin time and partial thromboplastin time (PTT)

Prothrombin time and partial thromboplastin time that also known as activated partial thromboplastin time (aPTT) levels were increased in patients with COVID-19. The results of 183 patients were evaluated in which higher prothrombin time and activated partial thromboplastin time were observed [64].

In a study, dynamic changes in d-dimer and prothrombin time indices were evaluated for prognosis of COVID-19. The level of all these factors was reported to be high in patients and these factors were valuable in the prognosis of the disease and mortality [78]. Prothrombin time and fibrinogen in severe COVID-19 patients were reported to be higher than those with mild [79]. In addition, shorter activated partial thromboplastin time and longer prothrombin time have been reported [80] and Prothrombin time has been low in patients with venous thromboembolism [81].

in a study, Prothrombin time and activated partial thromboplastin time were showed to be normal [82]. in another study, no relationship was found between prothrombin time, activated partial thromboplastin time and disease severity [83]. However, prothrombin time and C-reactive protein levels were high in ICU COVID-19 patients and they can be used as a biomarker to severity of the disease [84]. In addition, other coagulation abnormalities such as low antithrombin III, associated with a high risk of thromboembolic events [85].

## Inflammatory markers

### Neutrophil-to-lymphocyte ratio (NLR)

Neutrophil-to-lymphocyte ratio is a prognostic factor for hematological condition that this rate has been reported to be high in patients with COVID-19 [65]. It was reported higher levels of neutrophil-to-lymphocyte ratio in critical and severe COVID-19 patients [86].

A study was showed that the change of neutrophil-to-lymphocyte ratio is useful to determine the severity of COVID-19 and increased neutrophil to lymphocyte ratio was associated with mortality [66] and in a study of 245 patients with COVID-19, it was suggested that assessment of neutrophil-to-lymphocyte ratio was a good choice to identify high-risk person [87].

### C-reactive protein (CRP) and procalcitonin (PCT)

C-reactive protein is an acute phase protein and is high in severe COVID-19 patients that can be considered as a valuable biomarker [88]. Data from COVID-19 patients identified the role of C-reactive protein as a marker in predicting the possibility of exacerbation in non-severe patients [89].

A study of 76 patients with COVID-19 showed that high PCT and CRP levels were associated with mortality [90]. PCT is an inflammatory marker that has been used as an indicator for detection of relevant infections. Clinical manifestation such as PCT could indicate the progression of COVID-19 and higher serum PCT and CRP levels were observed in critical patients with COVID-19 [91, 92]. The high levels of C-reactive protein in people who died from infection indicated that it can be used as a biomarker to estimate disease lethality [93]. However, in another study, increase in serum C-reactive protein was associated with poor results in COVID-19 [94]. Prior to the detection of any change in Computed Tomography findings, C-reactive protein findings indicate lung destruction, which makes it a useful way in predicting severity in the early stages [95].

A study also showed that after treatment Patients with arterial thromboembolism and deep vein thrombosis, parameters, such as C-reactive protein and fibrinogen decreased [31].

### Lactate dehydrogenase

The higher level of lactate dehydrogenase is the freelance risk factors for exacerbation in mild COVID-19 patients [96]. The clinical features of COVID-19 patients showed that lactate dehydrogenase to be useful as a biomarker for early diagnosis of lung injury and severe cases of COVID-19 [97].

The relationship between lactate dehydrogenase and C-reactive protein concentrations with partial pressure of arterial oxygen to fraction of inspired oxygen ratio (PaO2/FiO2) indicated the efficiency of these two factors in the timely identification of patients with poor prognosis who require respiratory care and vital therapies [98].

In a study, the activities of daily living-dependency (ADL-dependency), high levels of d-dimers and lactate dehydrogenase, and the absence of anticoagulation were severally associated with 1-month mortality among older COVID-19 patients [99].

Finally, because the level of lactate dehydrogenase in severe COVID-19 shows a significant increase compared to the mild, serum lactate dehydrogenase level can be claimed as a useful parameter in assessing clinical susceptibility and follow-up of therapeutic response [100]. Receiving Pentoxifylline by patients was helpful in decreasing serum LDH [101].

### Antiphospholipid antibodies

Antiphospholipid antibodies abnormally target phospholipid proteins. A study was reported that antiphospholipid antibodies were temporary in COVID-19 patients [102]. Another study was also reported temporal antiphospholipid antibodies and found that high levels of aPLs in COVID-19 and genetically predisposed patients could lead to COVID-19-induced-antiphospholipid-like syndrome and must be controlled during the infection [103].

In addition, a study suggested a strong association between thrombosis and the presence of lupus anticoagulant (LAC), which is a criterion for antiphospholipid antibodies in severe COVID-19 and high prevalence of lupus anticoagulant in patients [104].

Finally, due to the low prevalence of antiphospholipid antibodies among COVID-19 patients with venous thromboembolism it can be suggested that antiphospholipid antibodies may not play a role in the pathogenesis of venous thromboembolism in severe COVID-19 pneumonia [105].

The number of patients with coagulation problems, changes in inflammatory and coagulation factors have been reported in Fig. 1.

### Cytokine storm

Overexpression of cytokines (IFN-γ, IL-1β, IL-2, IL-4, IL-6, and IL-10) have been observed in COVID-19 patients [1, 106]. Cytokine storm is elevated serum cytokine levels characterized by an uncontrollable inflammatory response associated with clinical deterioration of symptoms and mortality of COVID-19 patients [107, 108]. The relation exists between the generation of cytokine storm and ARDS development is directly associated with the mortality in COVID-19 [109].

A clinical trial was conducted on 102 patients with COVID-19 in Wuhan, China. The results showed higher serum level of cytokines, such as IL-6 and IL-10 in critical group than in moderate and severe ones, and these two factors can be used as predictors for fast diagnosis of disease deterioration [110]. In addition, high levels of IL-1β and IL-6 were observed in patients with severe COVID-19 than other patients [111].

Cytokine release syndrome (CRS) is a systemic inflammatory response that may cause by SARS-CoV-2 infection. IL-6 and tumor necrosis factor (TNF) have a pivotal role in the cytokine storm and can cause vascular leakage that leads to induce a hyper-coagulable status, which is a symptom of CRS [112–114]. A study showed that blockade of IL-6 can reduce COVID-19-induced CRS [115].

## Conclusion

Based on various studies, we found that coagulation problems, such as pulmonary embolism and venous thromboembolism, are common among COVID-19 patients and can lead to more vascular complications. Among coagulation markers, regular control of platelet count, especially d-dimer levels at admission and during hospitalization, can be helpful in early detection of coagulation problems. In addition, d-dimer is directly related to many coagulation problems and markers and can provide valuable information about mild to severe stages of the disease. Among inflammatory markers, we recommend regular monitoring of C-reactive protein. Besides these markers, following the onset of coagulation problems, nafamostat can be a better option for reducing these complications, compared to other drugs. Overall, these factors and measures can be effective in reducing coagulation disorders and improving patient care.

## Data Availability

Not applicable.

## Abbreviations

ADAMTS13: A disintegrin and metalloprotease with thrombospondin 1 repeats, number 13
ADL-dependency: Activities of daily living-dependency
aPTT: Activated partial thromboplastin time
ARDS: Acute respiratory distress syndrome
COVID-19: Coronavirus disease 2019
CRP: C-reactive protein
CRS: Cytokine release syndrome
DIC: Disseminated intravascular coagulation
DVT: Deep vein thrombosis
FDPs: Fibrin degradation products
FVIII: Factor VIII
HsCRP: High sensitivity C-reactive protein
ICU: Intensive care unit
IFN-γ: Interferon gamma
IL: Interleukin
LAC: Lupus anticoagulant
NCP: Novel coronavirus pneumonia
NLR: Neutrophil-to-lymphocyte ratio
PaO2/FiO2: Partial pressure of arterial oxygen to fraction of inspired oxygen ratio
PCT: Procalcitonin
PE: Pulmonary embolism
PLR: Platelet to lymphocyte ratio
PTT: Partial thromboplastin time
SARS: Severe acute respiratory syndrome
SIC: Sepsis-induced coagulopathy
TCP: Thrombocytopenia
TF: Tissue factor
TNF: Tumor necrosis factor
TPO-RAs: Thrombopoietin receptor agonists
Tα1: Thymosin alpha 1
VTE: Venous thromboembolism
VWF: Von Willebrand factor
